# Impact of Cross-Linking Procedure on Perioperative Quality of Life in Keratoconus Patients

**DOI:** 10.3390/jcm12113833

**Published:** 2023-06-03

**Authors:** Susanne Marx-Gross, Angelina Kroell, Daniel Wollschlaeger, Alexander K. Schuster, Jana C. Riedl, Joanna Wasielica-Poslednik, Norbert Pfeiffer

**Affiliations:** 1 Department of Ophthalmology, University Medical Center of the Johannes Gutenberg University Mainz, 55131 Mainz, Germany; 2 Artemis MVZ Wiesbaden, 65189 Wiesbaden, Germany; 3 Division of Pediatric Epidemiology, Institute for Medical Biostatistics, Epidemiology and Informatics, University Medical Center of the Johannes Gutenberg University Mainz, 55131 Mainz, Germany

**Keywords:** keratoconus, cross-linking, quality of life, EQ 5D5L, Nei VFQ 25, LFVFS, LFSES

## Abstract

Background: To evaluate the effect of crosslinking (CXL) with riboflavin for keratoconus (KC) therapy on quality of life (QoL): comparison of keratoconus patients with and without treatment. Methods: Prospective monocentric study. We recruited patients with progressive KC and with stable disease. Patients with progressive disease received cross-linking treatment; patients with stable disease were monitored. We compared QoL in both groups over 6 months and detected the influence of cross-linking treatment on QoL. QoL was assessed by NEI-VFQ-25, EQ-5D 5L, and EQ-Visual analog scale (VAS). In the evaluation of the Nei VFQ, the subgroups LFVFS and LFSES were calculated. Results: We enrolled 31 eyes of 31 patients in the intervention group and 37 eyes of 37 patients in the control group. Medians with standard deviations (SD) were calculated. All QoL-tests showed equal scores at baseline in both groups. At V2, one day after the treatment, EQ-VAS (56.4), LFVFS (57.4), and EQ5D5L (0.59) were significantly reduced. At V3 (one week after treatment), all results returned to baseline level. LFSES was not affected by the treatment. It remained stable (V2 85.4, V3 84.3). Comparing the baseline scores with the follow-up scores at month 6, we found a significant increase in QoL in all tests in the intervention group. Otherwise, the quality of life in the control group did not change over time. Conclusions: Cross-linking led only to a short-term reduction in QoL. Although the treatment is painful for a few days, no effect on general quality of life LVSES has been demonstrated. QoL already returned to baseline after one week and the patients were not limited anymore.

## 1. Introduction

Keratoconus is a non-inflammatory corneal disease, which progresses during adolescence and early adulthood. The irregularity and progressive thinning of the cornea can cause a refractive error, which cannot be easily corrected by glasses and leads to reduced vision. Keratoconus patients may use contact lenses to correct visual acuity, but in very advanced cases, only a corneal transplant is helpful. Currently, there are treatment options, such as corneal cross-linking, to stop the progression of keratoconus in an early stage [1].

Nevertheless, vision is often reduced and quality of life might be impaired [2]. Interacting with the environment relies heavily on processing the available visual information. Thus, visual impairment has an immense importance for most people. The deterioration of vision often makes adaptation of daily life and professional development necessary [3].

The cross-linking procedure is conducted as an out-patient intervention using topical anesthesia, which can be performed on adult patients as well as on children. The gold standard is the epi-off procedure according to the Dresdner protocol [1].

In this procedure, the epithelium is removed to allow the riboflavin to penetrate. This results in significant pain due to exposed nerve endings, which leads to a reduction in quality of life during postoperative care [4,5].

Furthermore, complications such as wound healing disorders (approximately 3–4%), epithelial hyperplasia and temporary stromal edema (approximately 1%) may occur in the early postoperative period. Rarely, these stromal edemas heal by scarring and lead to permanent visual deterioration. Especially in very steep corneas, hydrops may occur weeks to months later, leading to sudden visual decrease [6].

Both the early and the late post-cross-linking complications may affect vision and the quality of life.

Keratoconus patients often need rigid contact lenses for better and more distortion-free vision. However, no contact lenses should be worn on the affected eye within the first four weeks. Therefore, it is conceivable that after the wound has healed completely without complications and pain has been reduced, quality of life may still be impaired.

The purpose of our study was to assess the extent to which keratoconus and the cross-linking treatment cause impairment of quality of life. We are interested in the level and duration of the reduced quality of life and in possibly differential effects on separate aspects of quality of life.

## 2. Materials and Methods

Participants were recruited from the Keratoconus Center of the Department of Ophthalmology of the University Medical Center of the Johannes Gutenberg University Mainz, Germany.

### 2.1. Inclusion Criteria

Men and women of any ethnic background (>18 year) with progressive keratoconus undergoing cross-linking surgery;

Keratoconus patients with stable disease and no need of intervention were included as a control group;

Criteria for progression were the increase of Kmax more than 1 diopter (D) per year in Scheimpflug measurement axial/sagittal map and increase of more than 1 D in cylinder refraction.

### 2.2. Measurements

For the intervention group, the study visits were conducted before (V1: −28 to −1 days) and after the treatment (V2: +1 day postoperative, V3: +1 weeks, V4: +4 weeks, V5: +3 months, V6: +6 months). Visit 5 was conducted as a telephone call.

The patients in the control group participated in visits V1 and V6.

In both groups, the keratoconus diagnosis was based on Scheimpflug imaging (Pentacam^®^, Oculus, Wetzlar, Germany), Amsler grading and the Belin Ambrosio software. Patients were graded according to the Amsler classification system as Grades 1 to 4. We conducted one to three of these measurements until the quality was detected as “OK”, These measurements were made during V1 and V6.

In all cases, and at all visits except V5, a slit lamp biomicroscopy was performed and clinical findings of keratoconus such as stromal thinning, Fleischer ring, Vogt striae, and anterior stromal scars were detected and confirmed the diagnosis at baseline.

Glare sensitivity was detected by Mesotest II (Fa. Oculus, Wetzlar, Germany) and refractive error was analyzed by NIDEK^®^ AR 360-A (Fa. Nidek, Gamagori, Japan) at V1, V3, V4, and V6.

Best corrected visual acuity was tested by means at V1, V2, V3, V4, and V6 using number charts in 5 m distance. Contact lenses were not allowed because of the required break in wearing contact lenses prior to the examination.

Corneal thickness assessment was performed using the Pentacam^®^ (Fa. Oculus, Wetzlar, Germany) at V1 and V6.

All patients of the intervention group underwent a cross-linking procedure according to Dresden protocol: epithelium removal, application of Riboflavin 0.1% (MedioCROSS^®^ M and H, Avedro, Fa. Glaukos, San Clemente, CA, USA) solution over 30 min, 30 min UV-irradiance of 3 mW/cm^2^, 370 nm, minimal corneal thickness after epithelial removal 400 µm [1].

Postoperatively, patients were administered topical antibiotics (ofloxacin) and hyaluronic ointment (5×/d). After epithelial closure, they also received topical fluorometholone, initially given 5×/d and reduced over four weeks.

### 2.3. Quality of Life

To evaluate the patients’ quality of life, the German versions of NEI-VFQ-25 [7], EQ-Visual Analogue Scale, and EQ5D-5L [8,9] were administered to each participant of both groups. Participation in the study was proposed to the patient during V1, after all examinations and the consultation, the patient was then assigned to the appropriate group. After the patient’s consent, he or she filled out the questionnaire. During the follow-up visits, the questionnaires were always filled out before the consultation with the physician to avoid any influence of the results on quality of life.

The NEI-VFQ-25 includes 25 questions concerning quality of life and 13 special questions. The questions are divided into 12 subscales, which are general health, general vision and ocular pain, near vision, distance vision, social functioning, mental health, role limitations, dependency, driving, color vision, and peripheral vision [7].

During evaluation, each item is converted to a scale of 0–100. Each answer is attributed to a score that is correlated in relation to the patient’ level of impairment. The lowest score is 0 and the highest 100. A high value means better function or lower level of impairment. Instead of using the initially proposed subscales and total sum score of the NEI VFQ-25, we calculated the long-term visual functioning scale (LFVFS) and the long-term socioemotional scale (LFSES) on the basis of an analysis approach proposed by Pesudovs et al. [10]. The evaluation of these two scores was carried out in the same way as the calculations in the study by Nickels et al. [11]

The EQ5D-5L is a self-reporting health-related quality of life instrument, which makes it possible to determine a state-of-health classification followed by making a health evaluation using a visual analogue scale (EQ-VAS) [12]. The EQ-VAS is a measurement instrument used to detect the level of agreement for a special status such as vision. The participants rate their satisfaction on a scale from 0 (no agreement) to 100 (full agreement).

The descriptive EQ-5D-5L system defines so-called EQ5D-5L health states. A total of 3125 (=55) different health conditions can be recorded. Depending on the level, a number is assigned to each dimension, so that a five-digit number combination is obtained. This five-digit number can be converted into a point value using a special algorithm [13]. This score is known as the EQ-5D-5L index and represents the patient’s state of health.

### 2.4. Statistical Analysis

We used descriptive statistics to calculate measures of central tendency (medians, quartiles, means, standard deviations, and minimum and maximum values) for continuous variables (Quality of Life scores, visual acuity (decimal), and corneal thickness, Kmax).

Testing for differences in means of continuous outcomes between the intervention group and the control groups was done using *t*-tests for independent groups with Welch-corrected degrees of freedom. Testing for differences in means of continuous outcomes within the same group between two visits was done using *t*-tests for dependent samples. Non-inferiority of the intervention group compared to the control group for a continuous outcome at a specific visit was tested using the TOST (two one-sided *t*-tests) procedure.

*p*-values lower than 0.05 were regarded as statistically significant. Due to the exploratory nature of the study, we did not adjust *p*-values for multiple testing.

The analysis was carried out using the statistical R version 4.1.2 [14]. Statistical codes are available on request.

## 3. Results

In the intervention group, we enrolled 31 eyes of 31 patients who all received a cross-linking due to progressive keratoconus.

For control, we enrolled 37 eyes of 37 patients with a stabilized keratoconus. In the sample, 85% of the patients were below 40 years old. Most of the patients had have a moderate keratoconus level (Table 1).

At each visit, all subjects completed the three existing questionnaires and thus assessed their health status.

### 3.1. EQ-VAS

At baseline visit V1, the patients achieved a mean EQ-VAS of 81.7 ± 10.8 in the control group and 78.5 ± 15.2 in the intervention group (*p* = 0.3). In the intervention group, the EQ-VAS score was reduced to 56.4 immediately after the intervention (V2, *p* ≤ 0.0001), but rose back to the baseline score of 80.5 within the first days after treatment (V3, Table 2 and Table 3). EQ-VAS remained stable over the course of follow-up for four weeks to six months (V3–V6). A comparison between V1 and V3 shows no significant difference anymore (*p* = 0.4). Comparing the values from V1 to V6, there is a significant increase in visual satisfaction after six months (*p* = 0.01) (Table 3) However, no increase in visual acuity after six months could be observed in either group (Table 4).

In the control group, the EQ-VAS score of 81.7 ± 10.8 at baseline remained stable over the course of six months’ follow-up (79.7 ± 10.6) (*p* = 0.7) (Table 2 and Table 3).

### 3.2. Vision-Related Quality of Life (Nei VFQ 25)

The NEI-VFQ-25 test enables patients to assess their everyday needs. It signals the extent to which patients are restricted in their daily life.

Looking at the subscales of the test, lower scores were perceived in the subscale for general vision. Here, the baseline score in the intervention group was 58.9 and in the control group 62.3 for the male patients, and 62.9 and 63.3 for the female patients, respectively. In the intervention group, the patients rated their general vision as slightly better at the follow-up visit. In the baseline group, there was no difference.

We additionally calculated the LFVFS (long-form visual functioning scale) and the LFSES (long-form socioemotional scale) for every group and visit.

#### 3.2.1. LFVFS

Looking at the whole group, we detected a reduced score of LFVFS. The LFVFS at baseline was 75.2 in the control group and remained stable over the follow-up of six months (76.8) (Table 2 and Table 3).

The LFVFS of the intervention group was slightly lower at 67.7, compared to the control group at baseline (*p* = 0.1). The LFVFS score decreased to 57.4 immediately after the intervention (V2, *p* = 0.002), but rose to 69.3 after one week (V3). There was no significance between V1 and V3 (*p* = 0.9). After six months, the score rose to 77.9. Compared to baseline, there was a significant increase of LFVFS (*p* = 0.002) (Table 3).

#### 3.2.2. LFSES

LFSES was only slightly influenced by the intervention.

In the control group, we detected equal scores at baseline and at the six-month follow-up (91.1 vs. 89.1) (*p* = 0.4).

In the intervention group, we had similar scores (V1 87.7, V2 85.4, V3 84.3). In contrast to the LFVFS score, we detected no postoperative decrease.

However, we saw a significant increase at the six-month follow-up compared to baseline (*p* = 0.01) (Table 2 and Table 3).

### 3.3. EQ5D5L

The EQ-5D-5L score at baseline was in descriptive terms slightly higher in the control group compared to the intervention group (*p* = 0.1).

In the intervention group, the score (V2) was significantly reduced (*p* < 0.0001) immediately after the treatment, while no statistical difference compared to the baseline level was observed at V3 (one week after the treatment) (*p* = 0.6). At the six-month follow-up, we saw a significant increase compared to baseline. In the control group, the score rem ained stable over the six-month follow-up period (*p* = 0.1) (Table 2 and Table 3).

### 3.4. Other Parameters

All patients showed reduced vision at twilight, especially with glare. All nyctometer tests without glare showed medians of 0 (SD ± 1.6 test units), but the range was 0 to 1:2. At V3, however, all values were 0 (SD ± 0.8 test units). The glare test showed reductions throughout the collective group of patients, with a median of 0 (SD ± 1.5 test units).

The other parameters such as keratoconus grade, visual acuity, pachymetry and Kmax, which the study groups describe in more detail, can be found in Table 4, Table 5 and Table 6.

Last, we performed a correlational analysis of the level of association between questions 4 and 19 of the Nei-VFQ on pain and its impact on daily living and VAS, EQ5D-5L, LFSES, and LFVFS.

Some 90.3% of patients reported only mild to moderate pain, while 61.3% felt that none to only half of their daily activities were restricted by pain.

Furthermore, there were also no statistically significant correlations between the results of questions 4 and 19 and the other results of VAS, EQ-5D-5L, LFSES and LFVFS.

## 4. Discussion

Good visual acuity seems to be of enormous importance for the assessment of QoL, as vision-related quality of life depends highly on visual acuity. In addition, general measures of QoL are also reported to be related to visual function [15]. Mobility, e.g., driving a car, also depends on visual acuity.

Keratoconus patients usually develop the disease during adolescence or early adulthood. At this period of life, the person is very active, in the middle of professional life or is getting his or her education.

The knowledge that the disease may progress and that vision might decrease may also affect quality of life [16]. If surgery is then required due to documented progression, this may be additionally distressing. The immediate perioperative period with pain/foreign body sensation and reduced visual acuity at least will affect the patient.

This might lead to an incapacity for work. Furthermore, the patients are not allowed to wear contact lenses on the treated eye for approximately four weeks and therefore a further influence on the QoL can be assumed.

To shorten the painful period, we generally used an eye patch in the first postoperative days, which led to rapid healing of the epithelial defect and stopped pain [5].

In the present study, we analyzed QoL in keratoconus patients. For the first time, the influence of cross-linking on QoL was detected.

For this evaluation, we used different measurement tools.

Three tests had to be performed: the EQ-VAS, which is mostly influenced by visual impairment and the EQ5D-5L which detects the general QoL [17]. In the study, they were initially used to detect QoL in keratoconus patients.

The third test was the Nei VFQ 25. This test has often been used in different studies to detect QoL in keratoconus patients [2,18,19,20,21].

There are different strategies for interpreting the Nei VFQ 25 test. It is possible to evaluate and compare the 12 subscales individually. Using this approach, each individual question can be analyzed and compared. However, we chose an evaluation analogous to Pesudovs et al., in which the subscales are classified into a long-form visual functioning scale (LFVFS) and a long-form socioemotional scale (LFSES). Thus, the influence of vision will be considered separately only by analyzing the LFVFS [10].

In the present study, we analyzed the influence of cross-linking procedure on the visual QoL measured by EQ-VAS and LFVFS and the general QoL measured by EQ5D5L and LFSES in keratoconus patients.

To what extent and for how long are patients impaired?

Surprisingly, there is only a slight influence or none at all on general QoL, whereas the visual acuity-specific QoL is mainly the one affected. Based on the pain, one might assume that the overall QoL would also be affected.

However, the significant reduction in QoL on the first postoperative day was limited to the visual QoL.

### 4.1. Baseline and Keratoconus Disease

#### 4.1.1. EQ-VAS Score and LFVFS

EQ-VAS generally decreases with age and in case of chronic diseases i.e., heart disease, diabetes, and depression [9,22,23,24]. The influence of age on the EQ-VAS could be attributed to the increase of relevant diseases on the one hand, but also to the progressive decrease of visual acuity in old age caused by ophthalmic diseases on the other hand. Keratoconus disease can be classified as a severe disease, which impairs vision already at a younger age.

In our study group, we detected an EQ-VAS score of 81.7 for patients with stabilized disease and 78.5 of those with progressive disease. This is lower than the score of the normal population in a similar age group of 20–49 in Huber et al. They detected a score ranging from 87.1 to 95.7 [9] for a German population. Other research groups found an EQ-VAS score for the normal Bulgarian population between 80.4 and 89.7 for 18–44 years old, with younger people having higher scores [25]. This is more similar to our scores. This could be due to good therapeutic options such as visual acuity improving contact lens care.

Comparing the two groups of keratoconus patients in our study at baseline, we found that the group of those with stable disease had a slightly higher EQ-VAS score (81.7) than those with progressive disease and impending cross-linking (78.5). This effect has also been shown by other research groups such as Steinberg et al. [20], but was not significant (*p* = 0.3) in our study group.

Others, i.e., the population-based cohort study, the Gutenberg Health Study (GHS), by Nickels et al. determined the LFVFS and the LFSES scores of the Nei-VFQ 25 in the population of Mainz/Mainz-Bingen, Germany, and examined 15,000 persons [11]. The sample is representative of the population in Mainz/Mainz-Bingen and can be projected to that of all Germany.

Regarding the LFVFS score, in the GHS cohort, they detected higher scores (92.8 for male and 90.5 for female) compared to our results of 75.1/68.7 (male) and 76.1/74.3 (female) at baseline of our study. Therefore, the LFVFS 25 score seems to be influenced by keratoconus and the reduced visual acuity [11].

We assumed that the psychological effect of knowing about the progressive nature of the disease and the upcoming treatment leads to a slight decrease of the visual QoL at the baseline visit.

#### 4.1.2. EQ5D-5L and LFSES at Baseline

If we now consider the EQ5D5L as well as the LFSES of the Nei VFQ, which tests the general QoL, we find that in the baseline, there is no significant limitation of QoL due to keratoconus disease.

Considering the LFSES value, no influence of keratoconus disease is detected. Our values (89.8/86.0 (male); 100.0/96.2 (female)) are on the same level as in the GHS cohort (98.3 (male) 98.1 (female)) [11], and in part even higher.

The measurements of the general QoL by EQ5D-5L in keratoconus patients showed no impairment compared to that of healthy subjects or to that of patients with moderate glaucoma [25,26].

We found similar results in comparison to the Bulgarian cohort studies by the Encheva working group [25] at baseline.

The same results were obtained by the working groups of Hinz and Grochtdreis, who examined the normal German population with the EQ-5D-5L. In the 18–59 age groups, the sum scores were always above 90 [8,27] and comparable to our study.

It can therefore be assumed that the parameters of mobility, self-care, usual activities, pain/discomfort, and anxiety/depression are only slightly influenced by the disease of keratoconus if it is progressive. In our study, the influence is greater if the patient is female (m: 0.92; f: 87). Baseline scores of the control group (m: 0.94; f: 0.97) and the male intervention group (m: 0.92) were above 90.

### 4.2. Influence of Cross-Linking

EQ VAS and LFVFS as visual acuity-dependent parameters showed that treatment significantly reduced QoL.

We showed that the reduction existed only immediately after cross-linking. Already one week post-operatively (V3), the values were similar to those at baseline.

Patients obviously no longer feel impaired and can resume their daily activities. For the treatment, however, we used the epi-off procedure and this leads to a foreign body sensation in the first few days.

Pentacam analysis was performed at the six-month follow-up and the effect of the cross-linking was determined. It is possible that the patient’s knowledge that he or she has been treated and presumably that the disease is stabilized leads to higher patient satisfaction at six months and to a significant increase of EQ-VAS and LFVFS scores in the intervention group.

It is notable that there is also a significant transient reduction in general QoL in EQ5D5L, which was initially unaffected by the disease as such.

The EQ5D5L specifically queries mobility and pain. This could explain why the values for V3 (epithelial defect, pain, no car driving) are lower due to the intervention, while there is no general restriction due to the disease.

Especially looking at the LFSES at V2 (right after the cross-linking) and V3 (one week after the cross-linking), we see no decrease in either group (male: V2: 83.7, V3: 81.7; female: V2: 100.0, V3 99.0). Overall, female patients appear to have higher socio-emotional satisfaction than male subjects. This difference was statistically significant at baseline (V1) of both groups and at V6 in the intervention group (Table 3).

The LFSES measures socio-emotional status, which is obviously neither influenced by the disease nor by the treatment. There are no significant differences among V1, V2, and V3. The keratoconus does not seem to affect the general life situation. Other studies on QoL and pain, e.g., in male osteoporosis patients, also showed no reduced overall QoL [28].

According to other studies, ocular pain affects the general QoL as measured by the Nei-VFQ test [29]. Apparently, in our patient group, the postoperative pain perception was lower than expected after all. The majority of patients perceived only mild to moderate pain on the first postoperative day. This may explain why no influence on the general QoL was observed in our group.

### 4.3. Baseline vs. Six-Month Follow-Up

Obviously, the values in the treatment group continued to increase over the course of the six-month follow-up period, even though the baseline outcome was hardly affected and showed values comparable to the normal population. The knowledge of now being treated and having stopped progression seems to generate another significant improvement in QoL.

This statistically significant improvement in the QoL was detected in all three tests conducted (Table 3).

Other study groups detected a reduced QoL after three months in patients with progressive keratoconus measured by Nei-VFQ 25. After a longer follow-up (six months in our study), it is obvious that the patients’ anxiety is reduced, as other studies have also shown [30].

We attributed this rapid increase in QoL (V3), which was also demonstrated with the other tests (EQ-VAS, LFVFS, LFSES, and EQ5D5L), to the detailed patient information provided as well as the close supervision and close patient monitoring (daily checks until epithelial closure). Even the LFSES, which already showed high values in baseline, showed a significant increase.

In making comparisons to analyses of other groups that have not discovered any positive effect on cross-linking, this may be due to the shorter follow-up period (three vs. six months) [20].

The time when the QoL was surveyed could influence the assessment.

The patients filled out the questionnaire at the baseline after the examination and discussion, since this was where the study was included. During all other examination appointments, this was done during the time before the examination and discussion.

This circumstance could influence the assessment of the QoL, since the patients in the intervention group learned about the need for cross-linking. However, we saw no difference between control and intervention groups at baseline.

The first assessment of the success of the treatment was made during the six-month follow-up. This circumstance could lead to an improved assessment of the QoL. However, the questionnaire was filled out prior to the meeting, so that it is possible that only the relief of having had the treatment led to an improved QoL.

Filling out the questionnaire after the final discussion might have led to a further increase for the stabilized cases.

Overall, the results were surprising, in that the patients were hardly restricted in their normal activities by the disease. Furthermore, the cross-linking had only a short-term impact on QoL. After just one week, the restrictions had disappeared again and the patients were able to pursue their normal activities. Apparently, the presence of haze in a few cases and the prohibition of contact lens wear were less disturbing than expected. An effect on visual acuity through the effect of crosslinking is not to be expected in this short follow-up period. This effect can possibly be examined in more detail in a further study and a larger study cohort [31,32].

All patients were able to resume normal work activities or education after one week at the latest.

## 5. Conclusions

Visual acuity-specific analyses of QoL were reduced in keratoconus patients compared to the normal population. The keratoconus patient’s activity level appears to be limited based on visual acuity.

However, the overall QoL, independent of visual acuity, was not reduced compared to the normal population.

For patients with progressive disease who required cross-linking treatment, QoL increased significantly in all tests at the six-month follow-up. This was attributed to the now-stabilized disease and thus to the patients’ relief.

Cross-linking, which must be performed to stabilize progressive keratoconus disease, surprisingly appears to affect patients only during a brief period and only in visual acuity-specific aspects of life. Although the treatment is painful for a few days, no effect on general QoL has been demonstrated.

Thus, the patient’s inability to work is limited to about 1 week, unless they are dependent on contact lenses and unable to work without them.

## Figures and Tables

**Table 1:** Correlation of gender and keratoconus (KC) stage in both groups (Amsler classification).

	Gender	KC 1/1-2	KC 2/2-3	KC 3/3-4	KC 4
Intervention *n* = 31	female *n* = 7	1	5	1	0
	male *n* = 24	0	8	14	2
Control *n* = 37	female *n* = 6	2	2	2	0
	male *n* = 31	5	15	10	1

**Table 2:** Comparison of patients regarding gender with (Intervention I) and without cross-linking (Control C) in all tests: long-term visual functioning scale (LFVFS), long-term socioemotional scale (LFSES), EQ-5D-5L (European Quality of Life 5 Dimensions 5 Level Version), and EQ-VAS (European Quality of Life Visual Analog Scale) at different visits (V).

	LFVFS				LFSES				EQ-VAS				EQ 5D 5L			
	Male	Female	*p*	All	Male	Female	*p*	All	Male	Female	*p*	All	Male	Female	*p*	All
C *n* =	31	6		37	31	6		37	31	6		37	31	6		37
C V1	75.1	76.1	0.9	75.2	89.8	100.0	0.00004 *	91.1	81.8	80.8	0.9	81.7	0.94	0.966	0.4	0.95
C V6	77.9	72.4	0.5	76.8	88.0	94.3	0.2	89.1	81.1	73.8	0.3	79.7	0.931	0.922	0.6	0.93
																
I *n* =	24	7		31	24	7		31	24	7		31	24	7		31
I V1	68.7	64.3	0.6	67.7	86.0	96.2	0.05 *	87.6	78.2	79.3	0.9	78.5	0.92	0.87	0.6	0.90
I V2	58.5	54.3		57.4	83.7	100.0		85.4	56.6	55.8		56.4	0.61	0.516		0.59
I V3	68.3	72.3		69.3	81.7	99.0		84.3	80.9	79.3		80.5	0.91	0.929		0.92
I V4	70.3	75.5		71.6	90.2	89.3		90.0	82.6	85.3		83.2	0.94	0.96		0.94
I V5	71.6	79.2		73.5	85.5	82.3		84.9	84.6	78.0		83.0	0.94	0.956		0.94
I V6	78.7	75.9	0.6	77.9	92.0	98.5	0.03 *	93.5	86.2	80.7	0.1	84.6	0.96	0.884	0.3	0.94

V1: baseline, V2: +1 d, V3 +1 Week, V4: +4 Weeks, V5: +3 months, V6: +6 months. * Differences between subgroups are significant (*p* < 0.05).

**Table 3:** Comparison of patients with (Intervention) and without (Control) cross-linking in all tests: Comparison of the different visits regarding significance levels: long-term visual functioning scale (LFVFS), long-term socioemotional scale (LFSES), EQ-5D-5L (European Quality of Life 5 Dimensions 5 Level Version), and EQ-VAS (European Quality of Life Visual Analog Scale).

***p* =**		**V1 vs. V2 ^#^**	**V1 vs. V3 ^#^**	**V1 vs. V6 ^#^**
LFVFS	Control	n.a.	n.a.	0.1
	Intervention	0.008 *	0.9	0.002 *
LFSES	Control	n.a.	n.a.	0.4
	Intervention	0.5	0.3	0.01 *
EQ-VAS	Control	n.a.	n.a.	0.7
	Intervention	0.00003 *	0.4	0.01 *
EQ5D5L	Control	n.a.	n.a.	0.1
	Intervention	0.000001 *	0.6	0.01 *
***p* =**	**Intervention vs. Control V1 ^+^**		**Intervention vs. Control V6 ^+^**	
LFVFS	0.1		0.8	
LFSES	0.4		0.2	
EQ-VAS	0.3		0.09	
EQ5D5L	0.1		0.7	

^#^ Paired *t*-test, ^+^ Welch two sample *t*-test, * Differences between subgroups are significant (*p* < 0.05).

**Table 4:** Analysis of keratoconus patients with (Intervention) and without (Control) cross-linking regarding visual acuity BCVA.

	Gender	*n*	Mean	Med	Min	Max	SD
Control V1	Male	31	0.60	0.63	0.20	1.00	0.23
	Female	6	0.69	0.80	0.40	0.80	0.18
	All	37	0.61	0.63	0.20	1.00	0.22
Control V6	Male	31	0.59	0.50	0.16	1.00	0.23
	Female	6	0.57	0.50	0.40	0.80	0.15
	All	37	0.59	0.50	0.16	1.00	0.22
Intervention V1	Male	24	0.39	0.40	0.05	1.00	0.23
	Female	7	0.51	0.63	0.16	0.80	0.22
	All	31	0.41	0.4	0.05	1.00	0.23
Intervention V2	Male	24	0.21	0.16	0.05	0.63	0.16
	Female	7	0.18	0.10	<0.05	0.5	0.18
	All	31	0.20	0.16	<0.05	0.63	0.16
Intervention V3	Male	24	0.33	0.32	0.1	0.80	0.19
	Female	7	0.36	0.32	0.125	0.63	0.15
	All	31	0.34	0.32	0.10	0.80	0.18
Intervention V4	Male	24	0.41	0.40	0.10	0.80	0.224
	Female	7	0.371	0.40	0.20	0.63	0.139
	All	31	0.403	0.40	0.10	0.80	0.206
Intervention V5	Male	*)	*)	*)	*)	*)	*)
	Female	*)	*)	*)	*)	*)	*)
	All	*)	*)	*)	*)	*)	*)
Intervention V6	Male	24	0.377	0.32	0.05	0.80	0.22
	Female	7	0.683	0.80	0.32	0.80	0.18
	All	31	0.466	0.50	0.05	0.80	0.25

*) not applicable.

**Table 5:** Analysis of keratoconus patients with and without cross-linking regarding pachymetry (µm).

	Gender	*n*	Mean	Med	Min	Max	SD
Control V1	Male	31	471	483	345	555	48
	Female	6	459	477	381	501	43
	All	37	469	477	345	555	47
Control V6	Male	31	470	476	349	573	53
	Female	6	467	467	443	494	20
	All	37	470	475	349	573	48
Intervention V1	Male	24	438	443	375	528	43
	Female	7	485	471	453	541	33
	All	31	449	453	375	541	45
Intervention V6	Male	24	432	427	344	525	47
	Female	7	473	465	434	534	37
	All	31	444	445	344	534	47

**Table 6:** Analysis of keratoconus patients with and without cross-linking in Kmax (D).

	Gender	*n*	Mean	Med	Min	Max	SD
Control V1	Male	31	54.6	52.9	47.9	78.6	6.5
	Female	6	55.2	54.1	47.6	69.5	8.1
	All	37	54.7	53.2	47.6	78.6	6.7
Control V6	Male	31	54.6	52.9	47.5	79.0	6.5
	Female	6	53.2	51.9	49.3	58.3	3.7
	All	37	54.3	52.9	47.5	79.0	6.1
Intervention V1	Male	24	62.0	58.2	50.5	86.9	9.6
	Female	7	54.1	52.2	49.5	61.1	4.0
	All	31	60.2	57.8	49.5	86.9	9.3
Intervention V6	Male	24	61.8	59.3	49.4	82.3	9.9
	Female	7	54.0	52.0	49.3	61.9	4.9
	All	31	59.5	57.1	49.3	82.3	9.4

## Data Availability

The data presented in this study are available on request from the corresponding author. The data are not publicly due to data protection reasons.
